# Annual Circular of Geneva College

**Published:** 1849

**Authors:** 


### ARTICLE VIII.

*Annual Circular of the Medical Institution of Geneva College, Spring Course to Commence March 14th 1848.*

A change has been made, as we learn by this document, in the time of commencing the annual course of lectures in this Institution, so that the term will hereafter be during the sixteen weeks immediately following the second Wednesday of March.

An impression that had obtained to some extent, that the members of the faculty who are connected with the Buffalo University, designed retiring from their connection with this institution is corrected as "no such intentions exist."

